# Monolithically Integrated THz Detectors Based on High-Electron-Mobility Transistors

**DOI:** 10.3390/s25113539

**Published:** 2025-06-04

**Authors:** Adam Rämer, Edoardo Negri, Eugen Dischke, Serguei Chevchenko, Hossein Yazdani, Lars Schellhase, Viktor Krozer, Wolfgang Heinrich

**Affiliations:** 1 Ferdinand-Braun-Institut (FBH), 12489 Berlin, Germany; 2 Department of Information Engineering, Electronics and Telecommunications, Sapienza University of Rome, 00184 Rome, Italy; edoardo.negri@uniroma1.it; 3 Istituto per la Microelettronica e i Microsistemi, Consiglio Nazionale delle Ricerche, 00133 Rome, Italy

**Keywords:** room-temperature THz direct detectors, THz HEMT antenna integration, resistive mixing, on-chip antenna, TeraFET

## Abstract

We present THz direct detectors based on an AlGaN/GaN high electron mobility transistor (HEMT), featuring excellent optical sensitivity and low noise-equivalent power (NEP). These detectors are monolithically integrated with various antenna designs and exhibit state-of-the-art performance at room temperature. Their architecture enables straightforward scaling to two-dimensional formats, paving the way for terahertz focal plane arrays (FPAs). In particular, for one detector type, a fully realized THz FPA has been demonstrated in this paper. Theoretical and experimental characterizations are provided for both single-pixel detectors (0.1–1.5 THz) and the FPA (0.1–1.1 THz). The broadband single detectors achieve optical sensitivities exceeding 20 mA/W up to 1 THz and NEP values below 100 pW/$\sqrt{\mathrm{Hz}}$. The best optical NEP is below 10 pW/$\sqrt{\mathrm{Hz}}$ at 175 GHz. The reported sensitivity and NEP values were achieved including antenna and optical coupling losses, underlining the excellent overall performance of the detectors.

## 1. Introduction

The frequency range between 100 GHz and 1.5 THz, bridging the microwave and infrared frequency regions, is usually referred as *THz spectrum* and presents unique advantages for various application contexts, such as detection defects in tablet coating, industrial product inspection, spectroscopy for chemistry and astronomy, material characterization, security (weapons concealed under clothing), wireless communications and power transfer, and medicine (detection of cancer and caries) [1,2]. THz waves in this range can indeed penetrate non-conductive materials like plastics, fabrics, and ceramics while remaining non-ionizing and safe for biological tissues.

The potential of THz technology in medical diagnostics [3], security screening [4], and material inspection [5] and characterization [6,7,8] is not just exciting, but also intriguing, as it opens up a wide range of applications. In this frequency range, powerful electronic sources, such as solid-state oscillators and frequency multipliers, are already available, enabling the development of high-performance systems. THz direct detectors, which convert THz waves directly into electrical signals, are essential for real-time, high-resolution imaging without the need for complex intermediary steps [9]. They do not require local oscillators and their integration with antenna structures. Still, they have poorer sensitivity than the heterodyne methods and deliver no phase information. Schottky diodes are mainly used as sensitive room-temperature power detectors with limited array capabilities [10]. Many previous approaches to THz direct detection have been based on silicon field-effect transistors (FETs) [11,12]. III-V FET THz detectors have been demonstrated using GaAs FET devices [13] and GaN high electron mobility transistors (HEMTs) [14]. Results on THz detection using InP heterojunction bipolar transistor (HBT) were preliminary investigated in [15] with poor DC characteristics and published in [16] without specialized antenna structures and with limited sensitivity. A monolithically integrated one- or two-dimensional array of Schottky diodes has yet to be shown. The focal plane arrays demonstrated so far are based on silicon [17] and GaN technology [18]. Due to their limited sensitivities, the output currents from the individual THz detectors must be time-integrated, which dramatically reduces the readout speed. Passive imaging without illumination is possible with THz direct detectors, as demonstrated in [19]. However, to acquire a THz image with a size of 2.7 cm × 4 cm of a hand at room temperature with an uncooled broadband THz FET detector, it takes several hours with an integration time of 200 ms, a scanning spatial resolution of 1 mm, and a scanning speed of 1 cm^2^/100 s. Hence, increasing sensitivity is paramount for the applicability of large THz detector arrays. Therefore, to fully capitalize on the potential of this frequency range, there is an urgent and growing need to develop larger and more sensitive monolithic integrated detector chips. These larger detector arrays are not just a development, but a necessity that would significantly improve image resolution and enable faster scanning over broader areas, making THz imaging systems more practical for real-world applications.

In this paper, we show various implementations of FET-based direct THz detectors. All designs are based on resistive mixing. Narrowband and broadband single THz detectors, together with a THz focal plane array (FPA), were designed and measured. A GaN HEMT based on a monolithic microwave integrated circuit (MMIC) process was employed using transistors with only a transition frequency $f_{T}$ of 50 GHz.

All designs feature best-in-class detection properties at room temperature and the monolithic integrated detectors enable scaling to two-dimensional arrays, allowing for the fabrication of THz camera chips. All measurements do not include correction for lens losses, substrate losses, or antenna and lens mismatch, leading to pessimistic sensitivity and noise-equivalent-power (NEP) values. With the performance achieved in this paper, building powerful imaging THz systems in the frequency range up to 1.5 THz is made feasible.

This paper starts in Section 2 with basics of resistive mixing, indicating that our THz detectors are operating in the resistive mixing regime. The design of the individual THz detector and antenna structures are discussed in Section 3. Section 4 presents the realized individual detectors and the focal plane array. The measurement setup and corresponding results for both the single detectors and the FPA are presented in Section 5 and Section 6, respectively. Section 7 concludes the paper with a discussion of the results.

## 2. Resistive Self-Mixing

**Figure 1:**
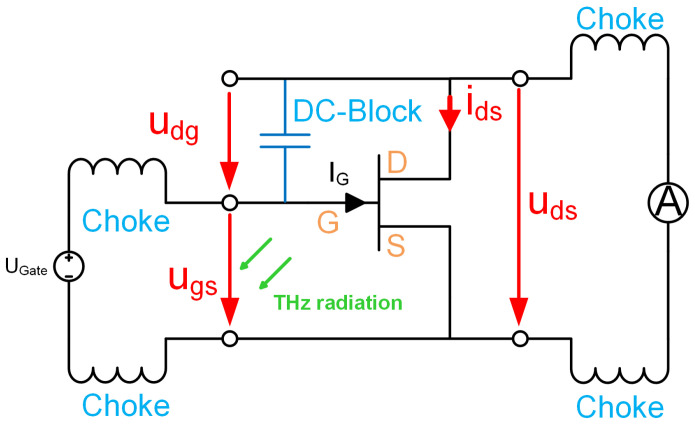
Schematic representation of the HEMT wiring for resistance mixing. The green arrows illustrate the THz radiation, which generates a voltage across the gate–source connection. The quantities represented through red arrows are the RF voltages and currents used for the THz detection.

With resistive mixing, the channel of an HEMT is used as a variable resistor. It is like using the same signal for the local-oscillator (LO) and radio-frequency (RF) signal in an FET-based mixer. The HEMT device works around the origin of the output characteristics [20] at zero drain–source voltage. To achieve this operating state in the THz detector, the THz radiation is fed between the gate and source connection, representing the LO signal. Through a broadband short circuit between the gate and drain connection, the same signal is present between the drain and source connection, which represents the RF signal. Figure 1 shows the equivalent circuit for resistive self-mixing.

The drain–source current $i_{ds}$ can be determined using a two-dimensional Taylor series expansion around an operating point with an arbitrary gate–source voltage and zero drain–source voltage. The transconductance of an HEMT $g_{m}$ as a function of gate–source voltage and the drain–source channel conductance $g_{ds}$ as a function of drain–source voltage

(1) $$\frac{\partial i_{ds}}{\partial u_{gs}}=g_{m}\;;\;\frac{\partial i_{ds}}{\partial u_{ds}}=g_{ds}$$

lead to a THz detection current at the drain terminals.

(2) $$\begin{array}{cc} i_{ds}\left(u_{gs},u_{ds}\right) & =g_{ds}\left(U_{GS},0\right)\Delta u_{ds}\\ & +\frac{\partial }{\partial u_{gs}}g_{ds}\left(U_{GS},0\right)\Delta u_{gs}\Delta u_{ds}\\ \; & +\frac{1}{2}\left\{\frac{\partial }{\partial u_{ds}}g_{ds}\left(U_{GS},0\right)\Delta u_{ds}^{2}\right\} \end{array}$$

The formula of the drain current in Equation (2) describes the drain current at a direct-current (DC) operating point where the drain–source voltage is zero and the gate–source voltage is a chosen parameter. Because of the RF short circuit, $\Delta u_{gs}\approx \Delta u_{ds}$, which results in

(3) $$\begin{array}{cc} i_{ds}\left(u_{sg},u_{ds}\right) & =g_{ds}\left(U_{GS},0\right)\Delta u_{gs}\\ \; & +\left\{\frac{\partial }{\partial u_{gs}}+\frac{1}{2}\frac{\partial }{\partial u_{ds}}\right\}g_{ds}\left(U_{GS},0\right)\Delta u_{gs}^{2} \end{array}$$

With a sinusoidal small-signal modulation between the drain and gate terminal, the THz detected drain current is

(4) $$\begin{array}{cc} \Delta u_{gs} & =A_{0}\sin\left(wt\right) \end{array}$$

(5) $$i_{ds}\left(u_{gs},u_{ds}\right)=\frac{1}{2}A_{0}^{2}\left(\begin{array}{c} \frac{\partial }{\partial u_{gs}} \end{array}+\frac{1}{2}\begin{array}{c} \frac{\partial }{\partial u_{ds}} \end{array}\right)g_{ds}\left(U_{GS},0\right)+\ldots$$

In Equation (5), only the DC component has been retained, and all higher-frequency components were discarded. In this relation, the differential output conductance at the operating point is highlighted in blue, the differentiation according to the drain voltage in green, and the differentiation according to the gate voltage in red.

The DC component depends quadratically on the THz voltage amplitude, $A_{0}$, which is determined by the source power and the antenna coupling. The second term in the parentheses describes the differential change in the output conductance as a function of the drain–source voltage (green). This represents the linearity of the differential output conductance. Since the drain–source bias voltage is zero at the selected operating point, this is the linearity of the differential conductance around the origin at the transition from the first to the third quadrant of the output IV characteristics. In a first approximation, this part can be neglected compared to the first term since, under small-signal conditions, the differential conductance at the origin represents a linear function of the drain–source voltage. The first term in the parenthesis is the change in the output conductance depending on the gate–source voltage (red), i.e., the ability of the HEMT to control the charge carriers in the channel.

**Figure 2:**
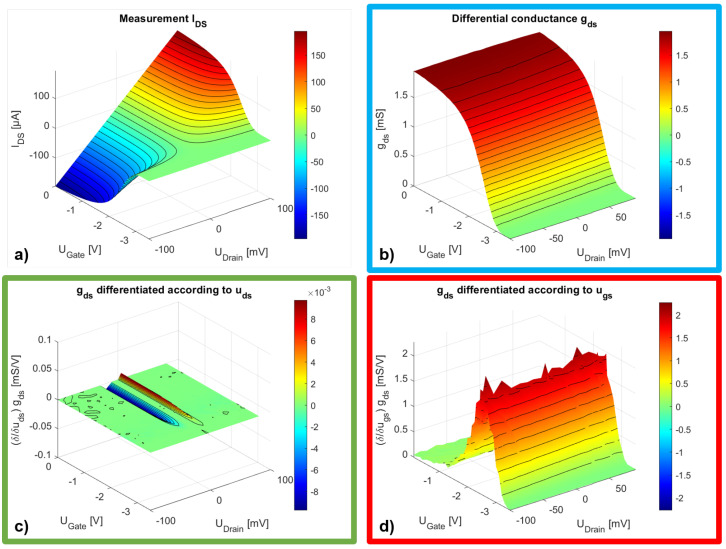
(**a**) High-resolution drain current measurement; (**b**) Differential output conductance; (**c**) Differential output conductance differentiated according to the drain voltage; (**d**) Differential output conductance differentiated according to the gate voltage.

The differential dependencies derived from Equation (5) can be represented using high-resolution DC measurements. To demonstrate this, we measured the behavior of the DC drain current as a function of the drain–source voltage and the gate–source voltage in steps of 0.5mV. Figure 2 shows the measurement result and the resulting differentials. The measurement was smoothed with an average filter to reduce noise. Otherwise, the minimal noise between the measured values will cause artifacts when forming the second derivative. The graphs show contour lines rather than individual measurement points to maintain clarity. Figure 2a shows the drain current measurement as a function of the gate–source and drain–source voltage. The gate–source voltage was varied between zero and −3.5 V, and the drain–source voltage between plus and minus 100 mV to capture the component’s behavior at 0 V drain–source voltage.

Even if the representation is somewhat unusual, it shows the behavior of a normally on component. We see the resistive control effect of the gate–source voltage up to the pinch-off at approximately −2.9 V and a linear dependence on the drain–source voltage. The graphs reported in Figure 2b–d represent the terms from Equation (5), where the colored frames correspond to the color of the equation’s terms. Figure 2b displays the measured differential output conductance $g_{ds}$, which primarily depends on the gate–source voltage. The graph in Figure 2c shows the differential conductance $g_{ds}$ differentiated concerning the drain–source voltage. As expected from the above discussion, the result is almost constant. In Figure 2d, the differential conductance is differentiated with respect to the gate–source voltage. The maxima show the gate–source voltage at which the largest differential change in the output conductance occurs. If the other term is neglected, this should be the gate–source voltage at which the resistive mixing causes the maximum detection current. We will review this consideration again in the Results Section and show the respective measured results.

From the result in Equation (5) and the graphical consideration, it can be deduced that the sensitivity of the THz detector structure depends on the properties of the HEMT and the antenna structure. The HEMT should have strong channel control, high charge carrier mobility, and low contact resistance. At the same time, the matching between the antenna structure and the HEMT should produce the highest possible gate–source voltage $\Delta u_{gs}$.

Based on this discussion, the THz detectors presented in the paper can be viewed as a voltage-controlled current source, with $\Delta u_{gs}$ as the control voltage.

## 3. Design

**Figure 3:**
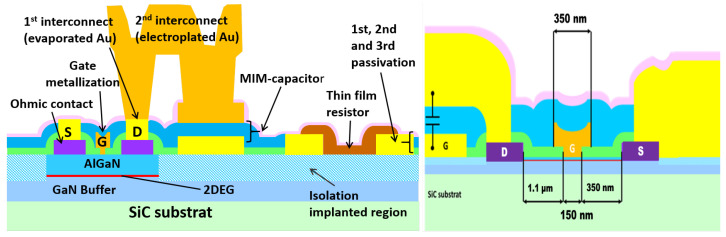
(**Left**): Stack of the used MMIC process. (**Right**): Schematic representation of the transistor with RF short circuit between the gate and the drain terminal.

We fabricated the THz detectors using our in-house GaN HEMT MMIC process, with the epitaxial layers of the heterostructure also grown internally. The epitaxial structure comprises an ${\mathrm{Al}}_{0.28}\mathrm{GaN}$ barrier layer with a thickness of 10 nm on a SiC substrate. A schematic representation of the MMIC stack is shown in Figure 3. The process incorporates two metallization layers, enabling the formation of metal–insulator–metal (MIM) capacitors. Silicon nitride, with a thickness of 200 nm, serves as the dielectric material. While the process also supports thin-film resistors, these were not utilized in the presented designs. The epitaxial layer structure and the specifics of the in-house MMIC process are described in detail in [21,22].

### 3.1. HEMT Dimensions

The HEMT transistors have the following dimensions:

- Gate length: 150 nm;
- Gate width: 3 µm and 5 µm;
- Gate head: 350 µm;
- Gate-source distance: 350 nm;
- Gate-drain distance: 1.1 µm.

Figure 3 (right) provides a schematic cross-section of the HEMT design, highlighting the short circuit between the gate and drain connections. The fabricated dimensions of the HEMT were determined by a trade-off between fabrication-related constraints, such as yield, and the need to minimize the size of the metallic structures at the antenna feed point. This design trade-off directly influenced the widths and spacing of the three terminals, requiring careful optimization to balance manufacturability with RF performance. The underlying design principle, explained using a specific detector design, applies uniformly to all the realized structures.

### 3.2. Design Requirements and Antenna Structure

**Figure 4:**
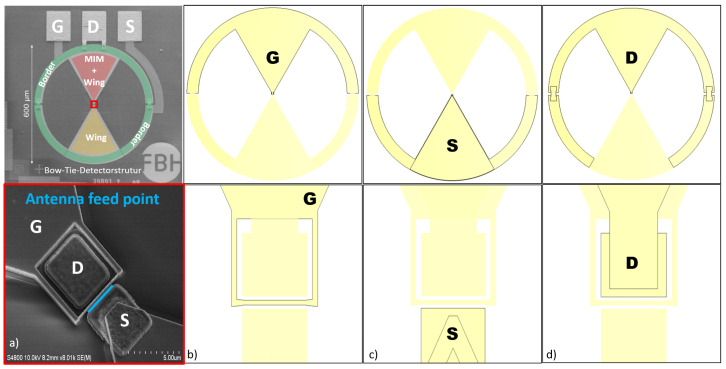
(**a**) Scanning electron images of a bow-tie detector structure and, on the bottom part, an enlarged image of the central HEMT. (**b**–**d**) Schematic representation of the galvanic connection of the three HEMT terminals.

As outlined in Section 2, the drain and gate connections must form an RF short circuit, while radiation between the gate and source connections should be introduced with high impedance. The antenna structure considered here is a planar bow-tie design with a 60-degree opening angle. Figure 4 illustrates the metal surface designs used to meet these boundary conditions and the achievable impedances. The antenna feed point between the gate and source terminals is marked in the first column of Figure 4.

- Top Row: Overview of the entire structure.
- Bottom Row: Detailed metallic structures of the HEMT.

The drain and gate terminals form an antenna wing, creating an RF short circuit over a wide bandwidth. The gate terminal acts as the lower electrode, and the drain terminal serves as the upper electrode of the MIM capacitor.

- Column (a): a scanning electron micrograph shows the partially fabricated HEMT, where the gate terminal completely encloses the drain terminal.
- Column (b): the continuation of the metal surface, directly connected to the gate terminal.
- Column (c): the metallic connection of the source terminal which is in the same plane as the gate connection.
- Column (d): the metallic connection structure of the drain terminal

Between columns (c) and (d), a 200 nm silicon nitride layer is applied over the surface, with openings at the drain terminal. Subsequently, the second metallization is vapor-deposited, contacting the drain terminal. MIM capacitances form where the metal layers overlap, as shown in the top row of Figure 4.

### 3.3. Integration of HEMT Terminals into the Design

The HEMT terminals directly form the RF environment, eliminating clear separation between the antenna and the HEMT device. All metallic connection structures are integral to the detector design, ensuring scalability to large arrays with minimal cross-coupling between individual elements. For frequencies above 100 GHz, the detector structures are designed as closed metal entities, with antenna wings merging seamlessly into the structural border. This approach:

- Prevents crosstalk with neighboring structures.
- Preserves antenna impedance by isolating connection structures.

**Figure 5:**
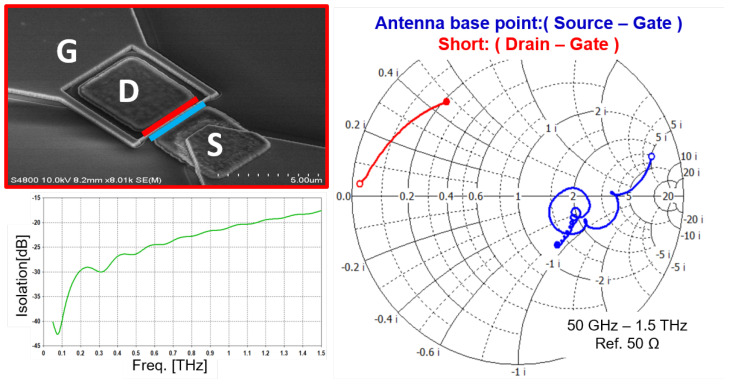
Simulation of the impedance (Smith chart on the right with a 50Ω reference impedance) and isolation (graph on the left in dB as a function of the frequency) between the drain–gate terminal and the source–gate terminal. The inset on the top-left corner represents in a color-coded manner the simulated reference points on a scanning electron image of the antenna feeding point.

Figure 5 presents the *S*-parameter simulation for two reference planes:

- Drain and gate terminals (red curve).
- Source and gate terminals (blue curve).

The simulation covers frequencies from 50 GHz to 1.5 THz, which correspond to substrate wavelengths ranging from 1.9 mm to 63 µm. The entire structure, including metallic surfaces and dielectric layers, was simulated using an electromagnetic (EM) full-wave solver (CST Microwave Studio).

As concerns the impedance behavior at the drain gate terminal, it is important to note the following:

- The real part of the impedance was less than 10 Ω over the entire frequency range.
- The reactance increases with frequency due to the inductive behavior introduced by the distance between the MIM capacitor and the reference plane.

In order to improve the performance, the design would have to be adjusted. The MIM capacitor should ideally encompass the drain terminal structure to mitigate inductive behavior. This design was excluded to avoid yield reductions from potential short circuits at metal edges.

The simulation of the antenna feed point (between source and gate terminals) reveals three distinct frequency ranges:

- *Low Frequencies* (<80 GHz): The structure is small with respect to the operating wavelength, causing the impedance to be influenced by the external environment rather than the antenna.
- *Transition Range* (80 GHz–400 GHz): The structure’s edge acts as a short circuit, allowing the antenna to dominate impedance. However, the wavelength remains too large for efficient radiation.
- *High Frequencies* (>400 GHz): The antenna structure reaches at least a quarter of the wavelength, where the impedance is determined by the radiation resistance in parallel with a capacitance determined by the metallic structures of the HEMT.

Based on this principle, various antenna structures, including slot antennas, can be designed with appropriate guidance of metallic layers. All structures were developed using three-dimensional (3D) EM simulations.

## 4. Implementation

The individual detectors and the FPA, developed based on the design idea outlined above, are presented below.

### 4.1. Single Detector

**Figure 6:**
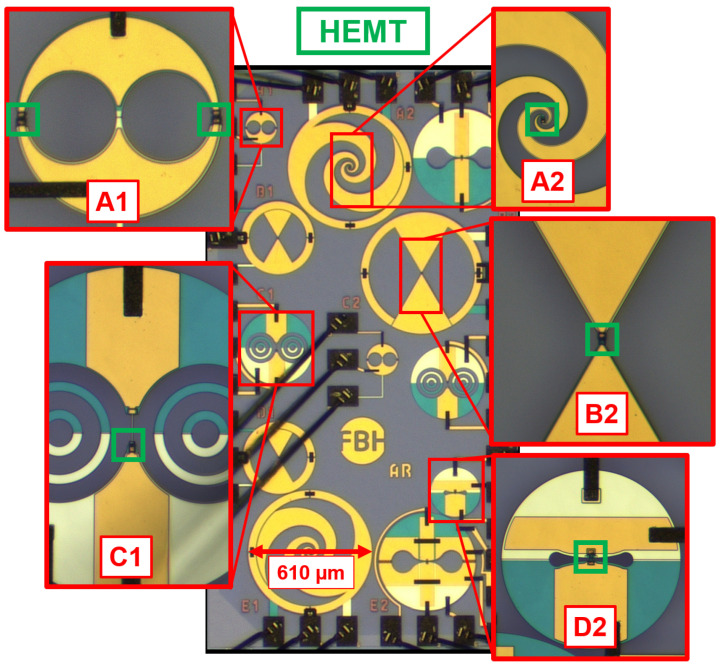
Photograph of the fabricated GaN HEMT THz detectors with various antenna structures and a close-up of the central structures, respectively. Green boxes highlight the HEMT structures on each detector.

Figure 6 displays a chip photo containing different THz detector structures with on-chip antennas. Enlarged views mark the HEMT integrations within the designs:

- Structures A2 and B2: Broadband THz detectors with log-spiral and bow-tie antennas, respectively;
- Structures A1, C1, and D2: Narrowband slot antennas covering 100 GHz to 400 GHz.

Unique features include:

- Structure A1: Two HEMTs connected in parallel at the structure’s edge.
- Structure D2: Narrowband design for 350 GHz.

The compact designs, particularly A1 and D2, are well-suited for future large THz focal plane arrays. For dimension comparison in Figure 6, it is essential to note that the diameter of the log-spiral structure is 610 µm.

### 4.2. THz Focal Plane Array

**Figure 7:**
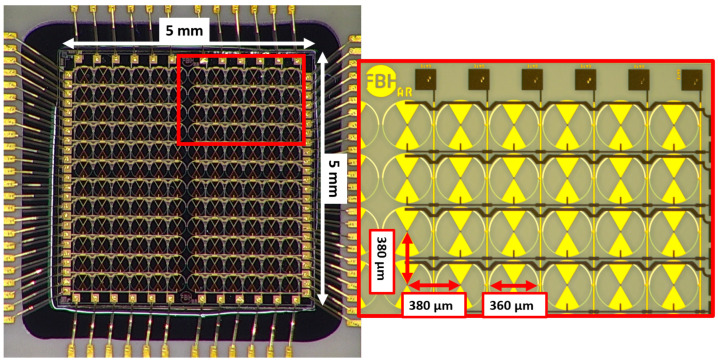
Image of the 144 pixel fabricated focal plane array of THz detectors, with a magnification of the first four rows in the first quadrant (inset on the right side).

The THz focal plane array chip measures 5 mm by 5 mm and features a bow-tie structure. Figure 7 presents an image of the manufactured and assembled focal plane array, along with a detailed view of the first four rows of the first quadrant. The chip has adhered to a highly resistive silicon substrate, through which the THz radiation couples.

The pixels are smaller than the individual detectors, measuring 360 µm in diameter compared to 610 µm. This relates only to the size of the structure; the guide of the metallic surface was executed according to the designs listed above. The detectors are arranged as follows: the entire focal-plane array consists of 12 × 12 detectors, totaling 144. This array is divided into four quadrants, each containing 6 × 6 detectors. The subdivision has the advantage that four pixels can be read out simultaneously; a detailed description can be found in Section 5. The distance from the center to the center of the detectors is 380 µm in both spatial dimensions. All detectors in a row share a common drain and source connection, indicating that they are connected in parallel. The detectors in a column share a common gate connection, which means that they are also connected in parallel with respect to their source connections.

## 5. Measurements

The same measurement setup was utilized for both the single-detector and array measurements. However, the evaluation electronics differed between the two analyses. The transmitter unit of a photomixer system (TeraScan 1550 [23]) was employed as THz power source. A silicon lens was used to couple the THz radiation into the SiC substrate (substrate illumination). The radiation from the THz source was focused onto a lens using two off-axis mirrors.

### 5.1. Single Detectors

The Keithley source measurement unit (SMU) 2600B was used to perform high-resolution DC measurements for each structure and, under THz radiation, generate the required gate–source voltage and measure the drain–source current, referred to as *detector current*. A motorized moving table placed the individual detectors into the beam focus. A calibrated pyroelectric THz detector (SLT Sensor und Lasertechnik; THz 20 HS) measured the THz power at the detector location. Since the SMU has a strong low-pass behavior, a chopping frequency of 10 Hz was used. The detection current was determined from the digitized signal using a fast Fourier transform (FFT).

### 5.2. THz Focal Plane Array

To evaluate a signal, the THz detectors arranged in a square grid are read out individually in a defined sequence with fixed addresses. Given the extensive range of current measured by the detectors, spanning from a few nA to twenty μA or more, an 18-bit analog-to-digital converter is employed. The low current necessitates a transimpedance amplifier with high gain. The layout of the THz focal plane array consists of four identical 6 × 6 detector sub-arrays. The detector features a gate-voltage-dependent sensitivity curve, as described in Section 5. A sufficiently high negative voltage at the gate switches off the GaN-HEMT detectors, causing them to behave like high resistance or an “open contact”. Setting the voltage to the optimal detection value enables the GaN-HEMT detectors to detect THz radiation. Within a sub-array, gates are connected in columns, while all drain and source terminals are connected in rows. Only one transimpedance amplifier is required per sub-array row.

To “read out” an individual detector, the optimal detection voltage must be supplied to the gate of that specific detector. Simultaneously, a sufficiently negative voltage must be supplied to all other detectors in the same row of that sub-array, ensuring that only the current from the chosen detector is sent to the connected transimpedance amplifier.

Dividing the entire detector array into sub-arrays offers several advantages. It enables the recovery of operational sub-arrays from otherwise dysfunctional whole arrays and facilitates an interleaved readout scheme for the detection currents, enhancing readout speed. After the currents of a particular column of a sub-array have been digitized, the GaN-HEMT detectors in that column must be closed. Subsequently, the next column of detectors must be activated to detect radiation by supplying the optimal detection voltage to those gates. This change, however, affects the currents in the drain and source pairs and, consequently, the net currents provided to the transimpedance amplifiers (TIAs). Due to the characteristics of high-gain TIAs, these devices have limited bandwidth and thus require some time to stabilize with the newly supplied detection currents, meaning digitization cannot occur during this settling time. Splitting the rows into two parts, enabled by the division into sub-arrays, allows for the connection of an additional, equal set of transimpedance amplifiers, which can provide voltages for digitization in the meantime. Interleaving the switching of columns in one set of sub-arrays with the digitization of currents in the other set allows for doubled digitization speed, making it the preferred approach for digitizing the detector current.

A more detailed examination of the readout electronics can be found at [18]. The readout speed of the current electronics is theoretically 100 frames per second (FPS). To improve the signal-to-noise ratio (SNR), dark current calibration was performed at the beginning of each measurement, followed by calculating an average across multiple recordings of the entire THz focal plane array, depending on the frequency and, thus, the power of the THz source. As the frequency rises, the averaging increases. At 100 GHz, only two frames were used, resulting in an FPS of 50 FPS. At 1.1 THz, 100 frames were utilized, which reduced the FPS to one.

## 6. Results

This section will present the measurements of the single detectors and the THz focal plane array. All measurements were obtained in the laboratory under standard atmospheric conditions at room temperature.

### 6.1. Single Detectors

**Figure 8:**
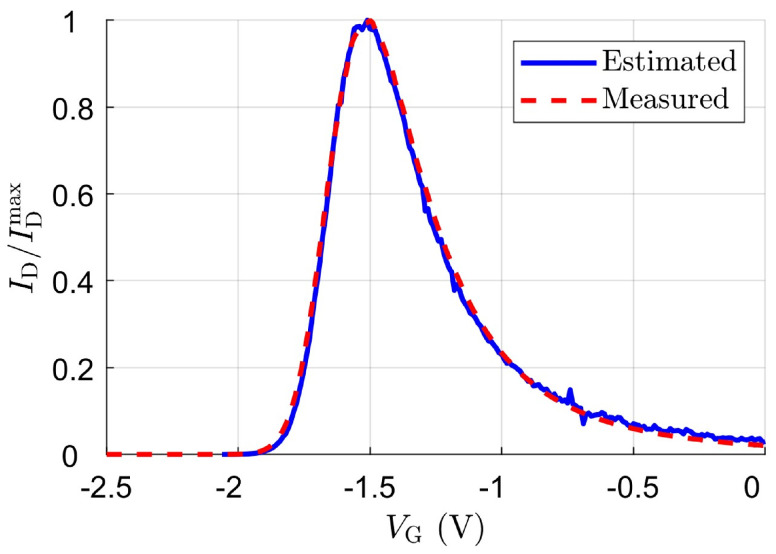
Determination of the optimal gate voltage using a high-resolution DC measurement. The theoretical estimation of the normalized drain current (blue solid curve) is an impressive agreement with measured results (red dashed line).

To verify the theory from Section 2, two measurements were carried out for each detector: a high-resolution DC measurement and a gate voltage sweep under THz radiation. The differential output conductance differentiated according to the gate voltage was computed using the DC measurement. The resulting plane was cut at the drain voltage equal to zero volts and superimposed on the result from the irradiation measurement. The result of these two measurements of the structure A2 is shown as an example in Figure 8. There is an excellent agreement between the maxima, so one can conclude that the theoretical derivation is valid. One can, therefore, predict the optimal gate voltage for resistive mixing with a theoretical analysis based only on a DC measurement.

**Figure 9:**
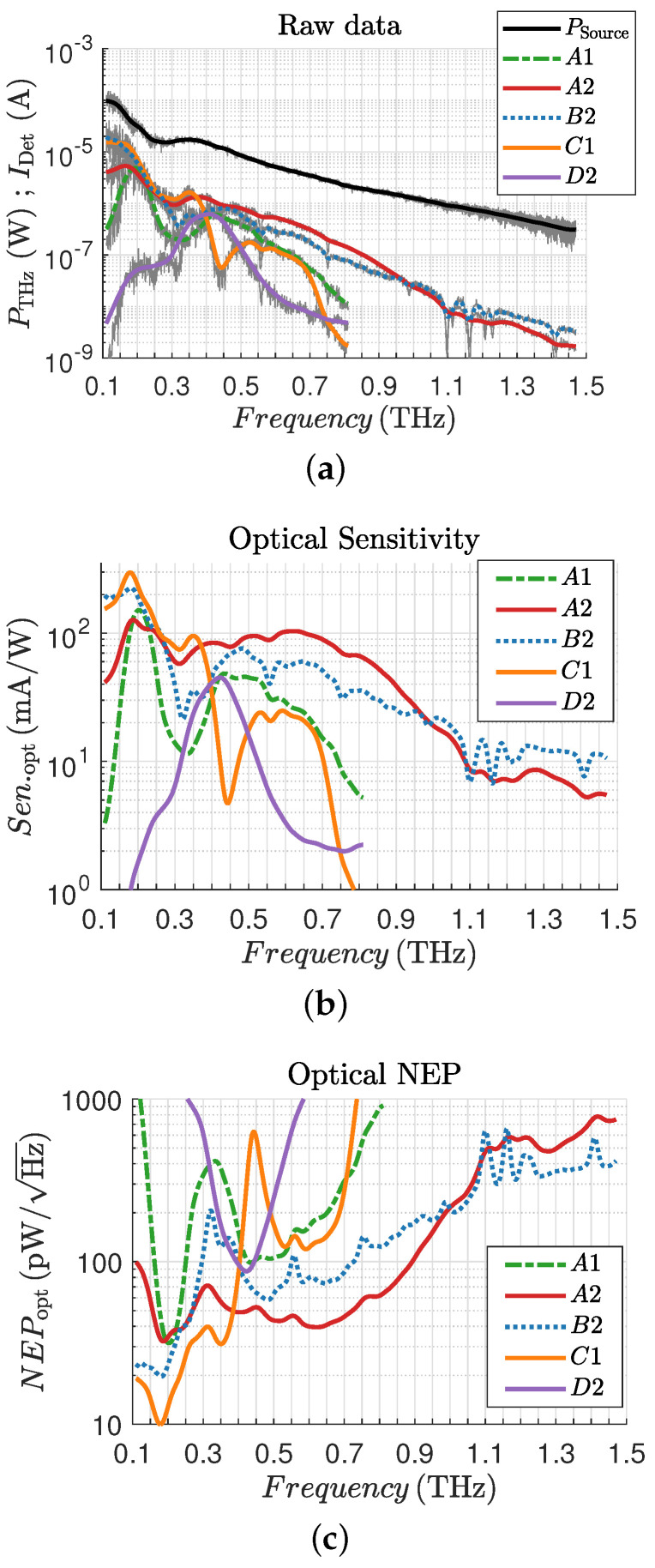
Measurement results: (**a**) raw data; (**b**) optical sensitivity; (**c**) optical NEP.

The optical sensitivity was determined using the measured detection current $I_{\mathrm{Det}}$ and the measured THz power $P_{\mathrm{THz}}$ at the detector location. The THz power and detection current were measured versus frequency with a step size of 1GHz. All raw data measurements and the smoothed curves are shown in Figure 9a. The measured frequency range depends on the design frequency. As can be seen from Equation (6) for the sensitivity employed here, we have ignored any losses of the lens and substrate or mismatches from the antenna, and we assumed that the antenna captures the complete power of the source, which provides a pessimistic (lower bound) value for the sensitivity.

(6) $$\begin{array}{c} Sen_{opt}=\frac{I_{\mathrm{Det}}}{P_{\mathrm{THz}}}\;\left[\frac{\mathrm{mA}}{W}\right] \end{array}$$

We determined the noise equivalent power for the individual detector structures based on this optical sensitivity $Sen_{opt}$ as

(7) $$\begin{array}{c} NEP_{opt}\;=\sqrt{\frac{4\;k_{B}\;T}{R_{V_{Gate\_opt}}}}\;\frac{1}{Sen_{opt}}\left[\frac{W}{\sqrt{\mathrm{Hz}}}\right] \end{array}$$

where $k_{B}$ is the Boltzmann constant, *T* is the temperature in Kelvin, and $R_{V_{Gate\_opt}}$ is the channel’s resistance at the gate voltage with the highest detection current. This calculation is based on the fact that the channel’s resistance is the noise source of the detector, which was confirmed in [24]. The average of the channel resistances of the five detector structures is 1.1 kΩ.

Figure 9b depicts the measured optical sensitivity and Figure 9c the optical NEP, determined using Equations (6) and (7) for all detector structures highlighted in Figure 6, respectively. The results in Figure 9 highlight in colors the mean values for the respective measurement. The THz power drops from 0.3 THz to 1.5 THz by two orders of magnitude, which is in accordance with the specifications for the THz source. The detected currents at the output from each individual detector are above 1 nA at all frequencies.

All THz detector structures show outstanding detector properties in terms of sensitivity and NEP. What should be highlighted here is structure A1, which occupies a significantly smaller area than the other THz detectors, but has a high sensitivity around 200 GHz. Both broadband detector structures A2 and B2 show a flat response up to frequencies around 0.8 THz and a drop in sensitivity beyond 0.8 THz. This drop is probably due to the gate width used (5 µm) and the associated capacitance in the antenna base. The capacitance connected in parallel to the antenna terminals causes the impedance to transform to lower impedance values, resulting in a smaller voltage swing. There are two clear extreme values for structure C1, a sensitivity maximum at 175 GHz and a sensitivity minimum at 400 GHz, which are probably related to a low feed point impedance and the far field of the antenna structure at 400 GHz. Structure D2 was designed for a frequency of 350 GHz and contains a clear minimum for the NEP and maximum for the sensitivity at 400 GHz with a 3 dB bandwidth of 200 GHz, which represents a shift of 50 GHz from the designed frequency. The lowest NEP below 10 pW/$\sqrt{\mathrm{Hz}}$ at 175 GHz is obtained for THz detector structure C1.

### 6.2. THz Focal Plane Array

**Figure 10:**
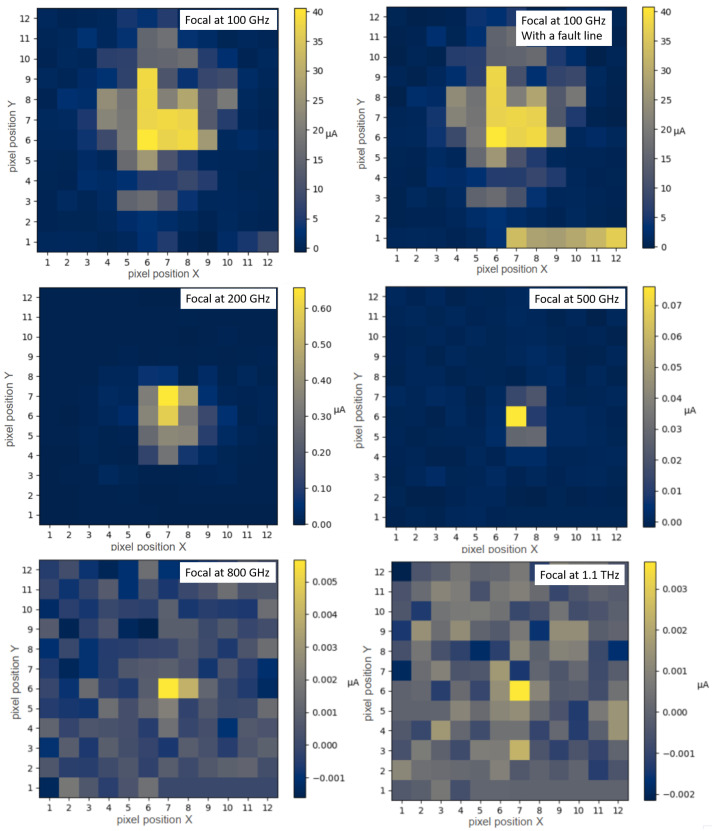
Measurement of the THz source focal spot from 100 GHz to 1.1 THz.

Figure 10 presents the initial test measurements with the THz focal plane array. We recorded the focal point at discrete frequencies within the range of 100 GHz–1.1 THz. A color bar next to the image represented the intensity recorded at each pixel and the resulting detection current. The images illustrate the result obtained after averaging several frames, as discussed in Section 5.2.

The upper row of the figure displays the measurement at 100 GHz. The right-hand figure illustrates the measurement with a defective line in the bottom row of the four quadrants. One detector malfunctioned, preventing the entire line from being accurately evaluated. This line was no longer considered in the subsequent measurements.

The second row presents the measurements at 200 GHz and 500 GHz, while the bottom row displays 800 GHz and 1.1 THz. As shown in the Figure 9a, the power of the THz source decreases exponentially with increasing frequency. This is directly reflected in the measurements despite calculating an average value over increasing frames. At 1.1 THz, which includes 100 frames, the SNR decreases noticeably, remaining just above 1.

## 7. Conclusions

We demonstrate THz detector designs using an AlGaN/GaN HEMT MMIC technology with excellent optical sensitivity and noise-equivalent-power performance. The underlying theory was derived and verified by measurement. Based on this theory, various detector designs and a focal plane array were realized and presented. The THz detectors were developed in a 150 nm GaN HEMT MMIC process. The monolithic integration of the HEMTs in various antenna structures is unique in design and enables a compact implementation in large arrays with minimal coupling between the array elements. We show experimental results for the single THz detectors and a focal plane array in the 0.1 THz–1.5 THz range. The detection mechanism presented here is not band-limited, and ultra-broadband designs with a bandwidth larger than 2 THz are possible. Only the antenna structures are indeed used to define the operating frequency and the bandwidth. The THz detectors have an optical sensitivity greater than 20 mA/W at 1 THz with an NEP < 100 pW/Hz. The lowest achieved optical NEP is less than 10 pW/Hz at 175 GHz. With a weak THz source, the resulting focal plane array demonstrates a real-time capability of 25 frames per second at frequencies up to 600 GHz.

All measurement results are the values obtained at the connector reference plane. These values can be used when designing a system, making the devices shown here suitable for building sensitive THz systems.

## 8. Patents

All THz detectors presented in this work follow the same design principle, which has been successfully patented in the EU, USA and Japan [25].

## Data Availability

The original contributions presented in the study are included in the article, further inquiries can be directed to the corresponding author.

## Abbreviations

The following abbreviations are used in this manuscript:

3D	Three-Dimensional
DC	Direct Current
EM	ElectroMagnetic
FET	Field-Effect Transistor
FFT	Fast Fourier Transform
FPS	Focal Plane Array
FPS	Frames per second
HBT	Heterojunction Bipolar Transistor
HEMT	High Electron Mobility Bipolar Transistor
LO	Local Oscillator
MIM	Metal Insulator Metal
MMIC	Monolithic Microwave Integrated Circuit
NEP	Noise Equivalent Power
RF	Radio Frequency
SiC	Silicon Carbide
SMU	Source Measurement Unit
SNR	Signal-to-Noise Ratio
TIA	TransImpedance Amplifier
